# Testing Antimicrobial Properties of Selected Short Amyloids

**DOI:** 10.3390/ijms24010804

**Published:** 2023-01-02

**Authors:** Przemysław Gagat, Anna Duda-Madej, Michał Ostrówka, Filip Pietluch, Alicja Seniuk, Paweł Mackiewicz, Michał Burdukiewicz

**Affiliations:** 1 Faculty of Biotechnology, University of Wrocław, Fryderyka Joliot-Curie 14a, 50-137 Wrocław, Poland; 2 Department of Microbiology, Faculty of Medicine, Wrocław Medical University, Chałubińskiego 4, 50-368 Wrocław, Poland; 3 Clinical Research Centre, Medical University of Bialystok, 15-089 Białystok, Poland

**Keywords:** antimicrobial peptides, amyloids, bacteria, machine learning, non-antimicrobial peptides, prediction

## Abstract

Amyloids and antimicrobial peptides (AMPs) have many similarities, e.g., both kill microorganisms by destroying their membranes, form aggregates, and modulate the innate immune system. Given these similarities and the fact that the antimicrobial properties of short amyloids have not yet been investigated, we chose a group of potentially antimicrobial short amyloids to verify their impact on bacterial and eukaryotic cells. We used AmpGram, a best-performing AMP classification model, and selected ten amyloids with the highest AMP probability for our experimental research. Our results indicate that four tested amyloids: VQIVCK, VCIVYK, KCWCFT, and GGYLLG, formed aggregates under the conditions routinely used to evaluate peptide antimicrobial properties, but none of the tested amyloids exhibited antimicrobial or cytotoxic properties. Accordingly, they should be included in the negative datasets to train the next-generation AMP prediction models, based on experimentally confirmed AMP and non-AMP sequences. In the article, we also emphasize the importance of reporting non-AMPs, given that only a handful of such sequences have been officially confirmed.

## 1. Introduction

Amyloid proteins (amyloids) are a diverse group of molecules that share little similarity in their amino acid compositions, but have the unique ability to assemble into filamentous aggregates characterized by the presence of cross β-structures [1]. These aggregates are typical of disorders called amyloidoses, including neurodegenerative diseases, such as Alzheimer’s (amyloid β, tau protein), Parkinson’s (α-synuclein), and Creutzfeldt–Jakob’s (prion protein—PrPSc) [2,3]. In addition to being linked to human pathological conditions, amyloids have also been reported to fulfill important biological roles, ranging from protection and storage to signaling and memory [4]. For example, they are the structural components of biofilms in bacteria, the reservoirs of proteins and peptides, e.g., hormones, in mammals, the elements of the primitive immune system in fungi, and most probably innate immune effectors against bacterial and virial infections in humans [4,5]. The propensity of a peptide/protein to form amyloid aggregates depends on the charge, hydrophobicity, and the β-conformation of aggregation-prone motifs. Amyloids can spontaneously self-assemble into fibrils; however, they may also trigger the aggregation of unrelated amyloidogenic proteins in the process known as cross-seeding [6,7]. Such interactions have already been observed in α-synuclein, amyloid β, PrPSc, and tau amyloid deposits in humans [6,8,9]. Research studies have not only shown that cross-seeding is possible between unrelated amyloidogenic proteins, but also between unrelated amyloidogenic proteins from different species [10,11,12,13,14,15].

Interestingly, amyloids share many similarities with antimicrobial peptides (AMPs), a diverse group of common molecules involved in the first line of host defence against microorganisms, as well as in microbial competition [16,17,18,19]. AMPs are short—generally up to 50 amino acids long—and do not display any consensus sequences, but share a positive charge, hydrophobicity, and amphipathicity [20,21,22]. Taking into account their secondary structures, AMPs can be grouped into α-helical peptides, β-sheet peptides, peptides with cross α—β structures, and extended linear peptides enriched in specific amino acids such as tryptophan, proline, and glycine [23]. AMPs exhibit many different mechanisms of action to trigger bacterial cell death, and consequently, it is much more difficult to develop resistance against them compared to traditional antibiotics [24,25,26]. Firstly, by interacting non-specifically with the anionic components of bacterial cell membranes, they damage the lipid bilayer by solubilization and/or pore formation [20,21,27]. Secondly, by acting intracellularly on specific molecules, they may inhibit proteases, cell division, and the biosynthesis of proteins, DNA, and RNA [28].

Similarly to AMPs, some amyloids have been shown to directly kill microorganisms by damaging their membranes. These characteristics have been reported for amyloid β [29], α-synuclein [30], and several other amyloids or their fragments, including tau [31], islet amyloid polypeptide [32], PrPSc [33], and endostatin [34]. As in the case of AMPs, monomers of amyloids self-assemble into oligomers and disrupt bacterial membranes by forming channels and/or acting like detergents, dissolving them. Interestingly, apart from destroying cell membranes by poration and solubilization, both amyloids and AMPs can kill bacteria by cell agglutination. This mechanism depends on their binding to bacterial wall carbohydrates, but it is still poorly understood [35,36].

The antimicrobial properties of amyloids have been especially well investigated in the case of amyloid β, which exhibits sequence and structural similarities with some validated AMPs [37]. Numerous studies indicate that amyloid β, in addition to its cytotoxic properties, may normally function as an AMP that is cleared after the inflammation subsides. However, when it becomes dysregulated, it forms toxic amyloid oligomers leading to neuronal cell death and eventually, to fibrillar deposits; the latter in turn trigger chronic inflammation [29,38]. Such a pattern of operation is exactly characteristic of AMPs, and it implies the existence of a delicate balance regulating antimicrobial versus cytotoxic properties for both amyloids and AMPs.

The next similarity between amyloids and AMPs lies in their propensity to form filamentous aggregates. This is not only restricted to amyloids, but is also characteristic of some AMPs. Protofibrils and fibrils have been proposed to entrap or agglutinate some pathogens, but their antimicrobial effectiveness is still debated [35]. Interestingly, certain AMPs are deposited as amyloids in common human amyloidopathies, including isolated atrial and senile seminal vesicle amyloidosis [5], but some AMPs have also been revealed to bind and inhibit amyloid aggregation [39,40]. The amyloidogenic properties have been ascribed to quite a few AMPs in vitro or in vivo, including lysozyme [41], protegrin-1 [42], plant defensins [43], HAL-2 [44], uperin 3.5 [45], Cn-AMP2, [46] or longipin [47].

The last discussed similarity between AMPs and amyloids is the fact that both can modulate the innate immune system. Their protofibrils and fibrils may directly bind to Toll-like receptors or self-assemble into ordered nanocrystalline complexes with immune ligands, such as DNA and dsRNA. These complexes mediate the amplification of inflammation via Toll-like receptors. Both AMPs and amyloids can engage a broad range of immune receptors, including TLR2, TLR3, TLR4, TLR9, FPR2, FPRL1, and NLRP3 (see [5] and the references therein).

Considering the substantial similarities between amyloid proteins and AMPs, we decided to verify the antimicrobial properties of short experimentally verified amyloids deposited in the WALTZ-DB 2.0 database [48]. We chose this particular group of amyloids because (i) their antimicrobial properties have not yet been investigated, and (ii) due to their length and resemblance to AMPs, they might potentially constitute a challenge for AMP prediction. The latter is of great importance for the development of the next-generation AMP classifiers, i.e., based on both experimentally validated AMP and non-AMP sequences [49]. To select the best candidates for experimental studies from the WALTZ-DB 2.0 database [48], we used AmpGram [50], a best-performing AMP prediction tool [49], and various other programs for comparative studies. The antimicrobial properties of the short amyloids were verified on ten bacterial strains representing both Gram-positive and Gram-negative species.

## 2. Results

### 2.1. Predicition of Antimicrobial Properties in Short Amyloids

The first stage of our research involved the selection of short amyloids with potential antimicrobial properties from the WALTZ-DB 2.0 database [48]. We approached this task with AmpGram, a bioinformatics tool based on n-grams (short amino-acid motifs) and random forests (a classification method in machine learning) [50]. We decided on this software because the first unbiased benchmark of AMP predictors clearly indicated it as the most accurate AMP classifier; it outperformed other models, independent of the dataset used for training and testing [49]. AmpGram’s superb performance might also suggest that short amino acid motifs are better at discriminating between AMP and non-AMP sequences than the global amino acid composition or physicochemical and structural properties commonly used by other AMP prediction models [49].

AmpGram [49,50] predicted ~6% amyloids deposited in WALTZ-DB 2.0 [48] as antimicrobial peptides, 32 sequences in total (Table S1), and from this pool, we selected the ten top predictions for our experimental research (Table 1). The ten short amyloids represented fragments of amyloid β, tau, PrPSc, α-crystallin B chain, or rationally designed sequences. The ~6% value seems rather low, considering the similarities between amyloids and AMPs. This indicates that (i) short amyloids are problematic for AMP predictors, e.g., they might be underpredicted, or (ii) these peptides do not have antimicrobial properties in general.

In order to cast some light on the prediction issue, we used an additional 15 AMP classification models to verify the AMP signal for the 10 amyloids selected for experimental research. Interestingly, only nine models (60%) predicted more than six amyloids (60%) as potential AMPs, and only one sequence VKIVYK (Amyloid 6) was predicted by all 15 methods. The inconsistent prediction pattern presented in Table 1 suggests that AMP predictors do exhibit some problems with the classification of short amyloids. Consequently, there are very interesting candidates for experimental research that are important from the point of view of developing AMP prediction tools.

### 2.2. Experimental Verification of Investigated Peptides in Terms of Amyloidogenic Properties

In the second stage of our research, we decided to determine the propensity of the ten short amyloids (Table 1) to form fibrils under the conditions routinely used to evaluate AMP properties, i.e., upon their incubation in Mueller–Hinton Broth (MHB; for details, see Materials and Methods). These experiments could also allow us to speculate whether the amyloid properties of peptides go along with their antimicrobial activities. To detect the presence of protein fibrils, we used a benzothiazole dye (thioflavin T) [51]. Thioflavin T binds to β sheet-rich structures in amyloid aggregates and triggers a characteristic red shift, i.e., an increase in the wavelength in both excitation and emission maximum of the dye [52]. Although all the investigated peptides were experimentally confirmed to be amyloids [48], only four formed aggregates in the MHB medium, namely: VQIVCK (Amyloid 1), VCIVYK (Amyloid 2), KCWCFT (Amyloid 5), and GGYLLG (Amyloid 8). The fluorescence spectra of the remaining peptides were indistinguishable from those of the medium itself (Figure S1).

### 2.3. Experimental Verification of Cytotoxic Properties of Short Amyloids

We also evaluated the antiproliferative influence of the ten short amyloids on the HEK-293 cell line (healthy human kidney cells) by measuring the activity of the oxidoreductase enzymes (MTT assay, see Materials and Methods) [53]. After 24 h exposure to human cells, none of the ten amyloids revealed cytotoxic effects on these cells. The IC_50_ values, i.e., the concentration that causes 50% growth inhibition of the cell line, were quite high, from 96 to 271 µg/mL (Table 2, Table S2). The slope and the coefficient of determination (R^2^) for all models used to calculate IC_50_ were statistically significant, with *p*-values < 0.003.

### 2.4. Experimental Verification of Antimicrobial Properties of Short Amyloids

**Figure 1:**
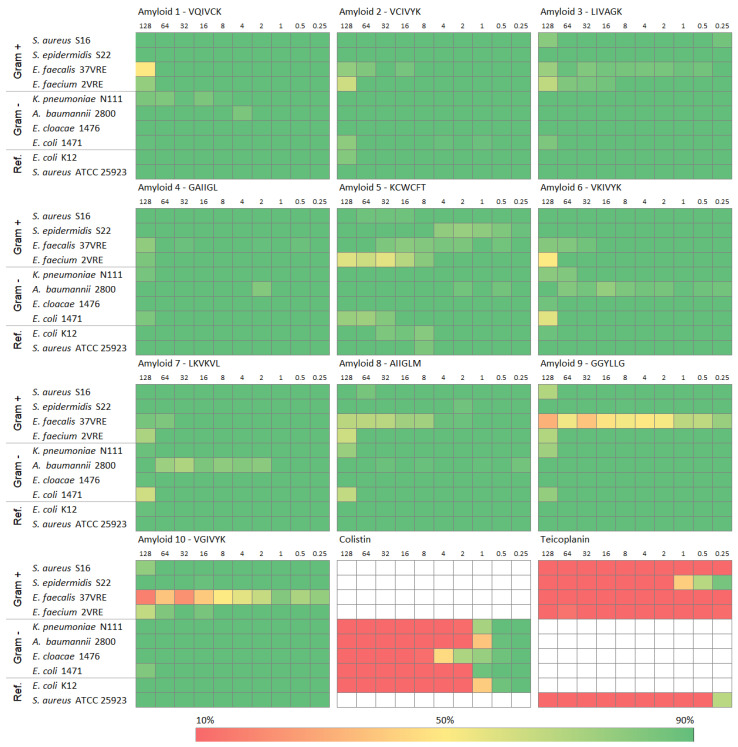
Experimental verification of the antimicrobial properties of short amyloids. Each of the 12 heat maps shows results for one amyloid or an antibiotic control. The columns represent the dilutions of the tested amyloids from 128 to 0.25 µg/mL, and the rows bacteria used, including Gram-negative (Gram-), Gram-positive (Gram+), and the reference species (Ref.). The effect of amyloids on the bacterial viability is indicated as a color gradient from red (less than 10% bacteria survived), through yellow (~50% bacteria survived), to green (more than 90% bacteria survived). The detailed results are presented in the Supplementary Materials.

In the final stage of our research, we verified the antimicrobial properties of ten short amyloids. We used ten bacterial strains, five Gram-positive and five Gram-negative, including eight clinical strains and two reference strains—100 combinations of peptides and bacteria in total (Figure 1, Table S3). As controls, we used colistin [54,55] and teicoplanin [56,57]. They have been used to treat multidrug-resistant infections, Gram-negative and Gram-positive, respectively. Both controls were proven to be very effective in destroying the tested bacteria; their MIC values, i.e., the minimum inhibitory concentration that inhibits visible growth (kills more than 90% of the inoculum within 18–24 h), ranged from 0.25 to 8 µg/mL, depending on the strain and antibiotic used (Figure 1).

The results presented in Figure 1 and Tables S4–S27 indicate that the ten short amyloids do not exhibit antimicrobial properties against Gram-positive or Gram-negative bacteria. We were not able to determine the MIC values within the 18 h incubation period for our peptide dilutions (MIC > 128 µg/mL), and accordingly, we were not able to determine their minimum bactericidal concentration (MBC), which is the lowest concentration of an antibacterial agent that kills 99.9% of the inoculum within 18–24 h.

The lowest viability (32% and 17%) was observed for samples containing *Enterococcus faecalis* 37VRE with GGYLLG (Amyloid 9) or VKIVYK (Amyloid 10), respectively, but in the case of the largest applied concentration of these peptides, i.e., 128 µg/mL. Importantly, this bacterial strain was characterized by fluctuating absorbance along the peptide concentration, which might result from the fact that it is very difficult to grow this strain in MHB, as it requires more enriched media (Table S12–S13 and Table S24–S25). Accordingly, the coefficient of variation (CV) for absorbance, defined as the ratio of the standard deviation to the mean, is often higher for *E. faecalis* than for the other tested bacteria. In the case of the samples with Amyloid 9 or Amyloid 10, CV is particularly high, as it ranges from 43% to 174% and from 55% to 300%, respectively. These values are much higher than the mean coefficient of variation calculated from the absorbance of all investigated bacteria for samples with Amyloid 9 (CV = 27%) and Amyloid 10 (CV = 32%).

Although the ten investigated amyloids do not have antimicrobial properties, our results for the regression analysis indicate that there is a significant negative correlation of bacterial viability with peptide concentration for 46 out of 100 bacteria–peptide combinations (Table S28). Accordingly, in some cases, there was some negative impact of the amyloids on the bacterial growth, even though the estimated MIC values were very high. The MIC lower than 512 µg/mL was found for only 22 cases of various combinations of amyloids, except for GAIIGL (Amyloid 4), with four bacterial strains: *Enterococcus faecalis* 37VRE, *Enterococcus faecium* 2VRE, *Klebsiella pneumoniae* N111, and *Escherichia coli* 1471 (Table 3). The most affected bacterium was *E. faecium,* with the lowest MIC of 177 µg/mL for VCIVYK (Amyloid 2).

## 3. Discussion and Conclusions

Given the substantial similarities between amyloids and AMPs, e.g., the propensity to kill microorganisms, form aggregates, and modulate the innate immune system, we decided to test the antimicrobial properties of short amyloids, as they have not yet been investigated. We used AmpGram, a best-performing AMP prediction model, and found 32 amyloids out of 509 to be potentially antimicrobial. In light of their substantial similarities, we thought at first that the number of amyloids predicted as AMPs might have been underestimated. Accordingly, we noticed that short amyloids are generally inconsistently predicted as AMPs or non-AMPs in comparative studies using 15 additional AMP classifiers (Table 1). This result indicates that short amyloids constitute a problematic group of peptides for AMP prediction.

From the pool of 32 potentially antimicrobial amyloids, we selected 10 with the highest AMP probability and determined whether they aggregate and negatively impact bacterial and eukaryotic cell growth. Four peptides: VQIVCK (Amyloid 1), VCIVYK (Amyloid 2), KCWCFT (Amyloid 5), and GGYLLG (Amyloid 8), formed aggregates in the MHB medium (Figure S1); however, none of the ten peptides were determined to be cytotoxic for eukaryotic cells (Table 2), nor did they exhibit antimicrobial properties against the tested bacteria (Figure 1, Table 3).

Our results indicate that the ten short amyloids should not be considered AMPs, due to their very weak impact on bacterial growth. Accordingly, they represent an immensely important group for AMP classification. In order to produce a reliable AMP prediction model, the developer must provide labeled data for its training and testing, i.e., a positive (AMP) and a negative (non-AMP) dataset. Shockingly, only 24 non-AMPs have been reported thus far, and all are deposited in the UniProt database (Table S29) [58,59]. Without having access to a good set of non-AMPs, the vast majority of developers working on AMP prediction have resorted to building the negative set from sequences deposited in public databases, including UniProt [59]. The procedure is based on sequence filtering options and clearly defined criteria, e.g., excluding sequences with antimicrobial properties and those possessing signal peptides (AMPs are mostly secretory proteins) from the dataset. However, the exclusion of peptides with assigned antimicrobial properties is not a good solution because the set could still include AMPs that have not yet been investigated. Consequently, the method is far from perfect, and it influences model performance [49].

Here, we present sequences for the negative dataset that are not only non-antimicrobial, but also very similar to AMPs. Consequently, they can be considered as adversarial examples that might substantially improve future AMP predictive models [60]. It is easy to develop a good machine learning tool to classify distinct sets, but this becomes more challenging when the sets contain more alike examples.

Given the growing interest in machine learning as the most cost-effective and the fastest method for discovering novel AMPs, addressing the issue of the flawed negative dataset is of paramount importance. We are absolutely positive that such data do exist, but are unpublished, being considered a side effect of many projects not worthy of any attention. At this point, we would like to strongly emphasize the significance of informing the scientific community about negative results—in our case, experimentally verified peptides that do not have antimicrobial properties. The systematic reporting of non-AMPs can help to construct a reliable negative dataset for AMP prediction.

## 4. Materials and Methods

### 4.1. In Silico Selection of Amyloids with Antimicrobial Properties

To choose the best candidates for our research, we downloaded experimentally validated short amyloids from the WALTZ-DB 2.0 database [48], with a total of 509 sequences. These amyloids were next analyzed for antimicrobial properties, with a modified implementation of AmpGram that worked on 5-mers instead of 10-mers to handle sequences shorter than ten amino acids [49,50]. The modified AmpGram has already been used with success in our previous studies of benchmarking AMP prediction tools [49]. For comparative studies, we chose 15 alternative AMP prediction models included in: ADAM [61], AI4AMP [62], AmPEP [63], Ampir [64], AMP Scanner Vr2 [65], CAMPR3 [66], Deep-AmPEP30 [67], IAMPE [68], MACREL [69], and AMPDiscover [70]; all were available as web servers. The computationally selected peptides were synthetized by CASLO ApS, with purities of >96%.

### 4.2. Strains and Culture Conditions

Eight hospital-isolated pathogens, four Gram-positive—*Staphylococcus aureus* S16, *Enterococcus faecalis* 37VRE, *Staphylococcus epidermidis* S22, *Enterococcus faecium* 2VRE—and four Gram-negative—*Klebsiella pneumoniae* N111, *Acinetobacter baumannii* 2800, *Escherichia coli* 1471, *Enterobacter cloacae* 1476—were used in the study (Table S2). Moreover, two reference strains, *Staphylococcus aureus* ATCC25923 and *Escherichia coli* K12, were used as representatives of non-pathogenic strains. The hospital-acquired strains were obtained from the collection of the Department of Microbiology at the Wroclaw Medical University.

All bacterial strains were stored at −80 °C. The strains were revived using overnight cultures prepared in Tryptic Soy Broth (TSB, OXOID, Basingstoke, UK) and incubated under shaking conditions (MaxQTM6000 incubator shaker, Thermo Scientific, Waltham, MA, USA) at 125 rpm and 37 °C. Next, the purity of the strains was evaluated using enriched media (appropriate for the tested strains). For each experiment, a fresh 18–20 h culture was prepared on Tryptic Soy Agar (TSA, OXOID, Basingstoke, UK) for Gram-positive bacteria and MacConkey Agar (MC, OXOID, Basingstoke) for Gram-negative bacteria, and the samples were then transferred to fresh Mueller–Hinton Broth (MHB, OXOID, Basingstoke, UK), where the density was determined. Each culture was established at 10^8^ CFU/mL and then diluted to a starting inoculum of 10^6^ CFU/mL.

### 4.3. Determination of the Antimicrobial Properties of Short Amyloids

The microdilution method was used to measure the antimicrobial activity of amyloids. The test was performed according to the recommendations of EUCAST (ISO 20776–1:2019).

In 96-well microtiter plates, geometric dilutions of the tested amyloids were prepared from 128 to 0.25 µg/mL in 50 µL MHB. The stock solution (20 mg/mL) of the synthesized amyloids was prepared in MHB. The bacterial suspension prepared in MHB with a density of 10^6^ CFU/mL was added to 50 µL of MHB containing the appropriate concentrations of amyloids, thus yielding a final bacterial concentration of 10^5^ CFU/mL. Pure MHB was used as a negative control, while the control for bacterial growth was MHB medium in the presence of the strain used in the experiment. Antibiotic solutions were used as positive controls: colistin for Gram-negative bacteria and teicoplanin for Gram-positive bacteria.

Microtiter plates were incubated at 37 °C for 18 h for *S. aureus*, *S. epidermidis*, *K. pneumoniae*, *E. coli*, *A. baumannii*, and *E. cloacae* and 24 h for *E. faecalis* and *E. faecium*. Optimal density (OD_600 nm_) was measured in a plate reader (ASYS UVM340, BIOCHROM Ltd., Cambridge, UK). The absorbance results were converted to percentages (with respect to growth control) in the normalization process. The MIC (minimal inhibitory concentration) for each peptide was defined as the concentration at which the destruction of at least 90% of the initial inoculum was observed.

Each experiment was performed in three independent studies for each of the two isolates, and each replicate was performed in three separate wells. In order to establish the MBC (minimal bactericidal concentration), 5 µL of the bacterial suspension from each well of the microtiter plate was inoculated onto a TSA plate and incubated for 24 h at 37 °C. The MBC value was defined as the lowest concentration at which no growth of the tested strain was observed.

### 4.4. Kinetics of Polymerization Process

The amyogenic abilities of the tested amyloids were determined in a kinetic reaction using the fluorescent dye thioflavin T (ThT), according to the modified method of Salinas et al. [71]. The peptide samples were dissolved in culture medium MHB (Ck = 20 mg/mL) immediately before the first measurement and mixed with an aqueous ThT solution (Ck = 20 µM). ThT fluorescence intensity readings were performed in 96-well opaque microplates using a SpectraMax GeminiXPS spectrofluorometer at an excitation of λ_ex_ 450 nm, an emission of λ_em_ 490 nm, and a cut-off point of λ_cut off_ 475 nm. Measurements were monitored for 24 h every 30 s. at 37 °C. The results were analysed in the SoftMax program. Fresh MHB culture medium was used as a negative control for all measurements.

### 4.5. Cell Culture

HEK-293 human embryonic kidney cells (ATCC, CRL-1573) were cultured in DMEM medium supplemented with 10% fetal bovine serum and 1% penicillin/streptomycin solution. The cells were cultured at 37 °C, 5% CO_2_, until the concentration was about 10^6^ per mL. A total of 2 × 10^5^ cells/mL were seeded in 96-well tissue microplates (2 × 10^4^ cells per well) and were allowed to adhere overnight under the conditions described above. Cytotoxicity tests were carried out on the cells prepared in this way.

### 4.6. Cell Proliferation Assays

Cells were incubated with varying concentrations from 128 to 0.25 µg/mL of the tested amyloid for 24 h exposure. The MTT assay has been previously described [72]. After incubating the cells with the tested amyloids, the MTT reagent (Ck = 0.5 mg/mL) was added to a well of a microtiter plate. After 2 h incubation (37 °C, 5% CO_2_), the MTT medium was removed and replaced with DMSO, to which was then added Sorensen’s buffer (0.1 M glycine, 0.1 M NaCl; pH 10.5). In the MTT test, the cell viability is directly proportional to the absorbance (570 nm). The control cells for background absorbance contained the growth medium, with MTT only. For MTT analysis, at least three experiments were performed using cells from consecutive passages.

### 4.7. Statistical Analyses

Linear regression analyses for viability of the HEK-293 cells subjected to ten amyloids, as well as the estimation of IC_50_ values, were determined in R Statistical Software using the model y = ax + 100% in the function lm from the stats package [73]. The regression analyses for the viability of the bacterial cells and the estimation of MIC were conducted based on a linear mixed-effects model y = ax + 100% + (1|isolate/replicate) in lmer function from R package lme4 [73]. Isolates and replicates were assumed as random effects. The *p*-values of the determination coefficient and the slope were corrected using the Benjamini–Hochberg procedure for the obtained models.

## Figures and Tables

**Table 1:** Classification of 10 amyloids, indicated by AmpGram with the highest AMP probability, using 15 alternative AMP prediction models. Values greater than 0.5 were colored in green and indicate potential AMP properties. Models marked with asterisks (*) produced zero-one outputs, without giving the probability. Citations for the models are provided in the Materials and Methods section. Amy = Amyloid.

AMP Prediction Model	Peptide Name and Its Sequence									
	Amy 1	Amy 2	Amy 3	Amy 4	Amy 5	Amy 6	Amy 7	Amy 8	Amy 9	Amy 10
	VQIVCK	VCIVYK	LIVAGK	GAIIGL	KCWCFT	VKIVYK	LKVKVL	AIIGLM	GGYLLG	VGIVYK
AmpGram	0.685	0.720	0.769	0.809	0.849	0.684	0.632	0.633	0.652	0.665
ADAM-SVM *	1.000	1.000	1.000	1.000	1.000	1.000	1.000	1.000	1.000	1.000
AI4AMP	0.686	0.174	0.292	0.280	0.292	0.564	0.254	0.534	0.181	0.553
AmPEP *	0.000	0.000	0.000	1.000	1.000	1.000	1.000	0.000	0.000	0.000
Ampir	0.350	0.452	0.658	0.636	0.185	0.767	0.611	0.744	0.578	0.322
AMP Scanner Vr2	0.774	0.869	0.331	0.747	0.890	0.753	0.357	0.760	0.579	0.737
CAMP3-ANN *	1.000	1.000	1.000	1.000	1.000	1.000	0.000	1.000	1.000	1.000
CAMP3-DA	0.086	0.449	0.593	0.081	0.781	0.880	0.811	0.001	0.264	0.405
CAMP3-RF	0.452	0.507	0.492	0.383	0.378	0.569	0.556	0.421	0.296	0.477
CAMP3-SVM	0.000	0.000	0.001	0.000	0.000	0.876	0.024	0.000	0.000	0.000
Deep-AmPEP30 *	0.000	1.000	1.000	1.000	1.000	1.000	1.000	1.000	0.000	1.000
IAMPE *	1.000	1.000	1.000	1.000	1.000	1.000	1.000	1.000	1.000	1.000
MACREL	0.505	0.386	0.535	0.455	0.257	0.574	0.743	0.426	0.069	0.446
RF-AmPEP30 *	1.000	1.000	1.000	1.000	1.000	1.000	1.000	1.000	1.000	1.000
RF-AMPDiscover	0.780	0.920	0.960	0.800	0.970	0.850	0.960	0.660	0.870	0.780
RNN-AMPDiscover *	1.000	1.000	1.000	1.000	1.000	1.000	1.000	1.000	0.000	1.000

**Table 2:** Parameters of regression analyses for viability of the HEK-293 cells subjected to ten amyloids and IC_50_ values calculated on the basis of the obtained models. R^2^ states for coefficient of determination.

Amyloid	Sequence	R^2^	*p*-Value	Slope	IC_50_ [µg/mL]
Amyloid 1	VQIVCK	0.563	3.93 × 10^−6^	−0.299	167
Amyloid 2	VCIVYK	0.500	2.04 × 10^−5^	−0.426	117
Amyloid 3	LIVAGK	0.350	0.00054	−0.423	118
Amyloid 4	GAIIGL	0.711	2.62 × 10^−8^	−0.433	116
Amyloid 5	KCWCFT	0.494	2.04 × 10^−5^	−0.523	96
Amyloid 6	VKIVYK	0.372	0.00039	−0.447	112
Amyloid 7	LKVKVL	0.271	0.00263	−0.184	271
Amyloid 8	AIIGLM	0.347	8.43 × 10^−5^	−0.337	148
Amyloid 9	GGYLLG	0.464	3.93 × 10^−6^	−0.292	171
Amyloid 10	VGIVYK	0.270	0.00054	−0.409	122

**Table 3:** Parameters of regression analyses for viability of the bacterial strains subjected to ten amyloids and the MIC values calculated on the basis of the obtained models lower than 512 µg/mL. R^2^ states for coefficient of determination.

Amyloid	Species	R^2^	*p*-Value	Slope	MIC
Amyloid 2	*E. faecium* 2VRE	0.516	1.4 × 10^−6^	−0.509	177
Amyloid 6	*E. faecium* 2VRE	0.465	4.9 × 10^−11^	−0.504	179
Amyloid 10	*E. faecalis* 37VRE	0.598	4.0 × 10^−5^	−0.465	193
Amyloid 1	*E. faecium* 2VRE	0.381	1.0 × 10^−4^	−0.460	196
Amyloid 9	*E. faecium* 2VRE	0.457	6.3 × 10^−8^	−0.440	204
Amyloid 1	*E. faecalis* 37VRE	0.645	3.8 × 10^−17^	−0.395	228
Amyloid 5	*E. faecium* 2VRE	0.425	1.0 × 10^−14^	−0.391	230
Amyloid 8	*E. faecium* 2VRE	0.446	1.2 × 10^−4^	−0.322	280
Amyloid 10	*E. faecium* 2 VRE	0.288	1.5 × 10^−4^	−0.305	295
Amyloid 3	*E. faecium* 2VRE	0.419	9.3 × 10^−8^	−0.299	301
Amyloid 6	*E. coli* 1471	0.500	2.9 × 10^−17^	−0.282	319
Amyloid 7	*E. faecium* 2VRE	0.316	7.0 × 10^−4^	−0.276	326
Amyloid 7	*E. coli* 1471	0.389	2.5 × 10^−11^	−0.261	345
Amyloid 2	*E. faecalis* 37VRE	0.554	4.1 × 10^−7^	−0.247	364
Amyloid 8	*E. coli* 1471	0.362	2.0 × 10^−14^	−0.242	372
Amyloid 9	*E. faecalis* 37VRE	0.641	4.9 × 10^−3^	−0.232	389
Amyloid 5	*E. coli* 1471	0.432	1.8 × 10^−17^	−0.222	405
Amyloid 9	*K. pneumoniae* N111	0.436	4.5 × 10^−12^	−0.221	406
Amyloid 7	*E. faecalis* 37VRE	0.308	8.7 × 10^−3^	−0.215	418
Amyloid 8	*K. pneumoniae* N111	0.355	1.8 × 10^−12^	−0.189	475
Amyloid 6	*K. pneumoniae* N111	0.375	3.0 × 10^−12^	−0.187	482
Amyloid 8	*E. faecalis* 37VRE	0.384	5.6 × 10^−5^	−0.182	496

## Data Availability

The data presented in this study are available in supplementary materials.

## Supplementary Materials

The following supporting information can be downloaded at: https://www.mdpi.com/article/10.3390/ijms24010804/s1.
