# Supplementary material for: Testing Antimicrobial Properties of Selected Short Amyloids
Source: Int J Mol Sci. 2023 Jan 2;24(1):804. doi: 10.3390/ijms24010804 (PMC9821130; doi:10.3390/ijms24010804)
Supplement: Supplementary file 1 [file ijms-24-00804-s001.zip › ijms-2088855-supplementary.pdf]

International Journal of *Molecular Sciences*

MDPI

Supplementary Materials

Testing Antimicrobial Properties of Selected Short Amyloids

Przemysław Gagat [1,*,†], Anna Duda-Madej [2,†], Michał Ostrówka [1], Filip Pietluch [1], Alicja Seniuk [2], Paweł Mackiewicz [1] and Michał Burdukiewicz [3,*]

1 Faculty of Biotechnology, University of Wrocław, Fryderyka Joliot-Curie 14a, 50-137 Wrocław, Poland
2 Department of Microbiology, Faculty of Medicine, Wrocław Medical University, Chałubińskiego 4, 50-368 Wrocław, Poland
3 Clinical Research Centre, Medical University of Bialystok, 15-089 Białystok, Poland
* Correspondence: przemyslaw.gagat@uwr.edu.pl (P.G.); michalburdukiewicz@gmail.com (M.B.)
† These authors contributed equally to this work.

Table S1. The list of 32 short amyloids from the WALTZ-DB 2.0 database (Louros et al. Nucleic Acids Research 2020, 48, D389–D393) predicted as potentially antimicrobial with AmpGram (Burdukiewicz et al. International Journal of Molecular Sciences 2020, 21, 4310; Sidorczuk & Gagat et al. Briefings in Bioinformatics 2022, 23, bbac343).

No.	Amyloid sequence	AMP probability	No.	Amyloid sequence	AMP probability
1	KCWCFT	0.8487997	17	NKGAII	0.5827486
2	GAIIGL	0.8094537	18	VHIVYK	0.5812274
3	LIVAGK	0.7687570	19	CGVIGI	0.5775986
4	VCIVYK	0.7203234	20	VWIVYK	0.5773327
5	VQIVCK	0.6846011	21	KQIGII	0.5771180
6	VKIVYK	0.6839378	22	GYCFIL	0.5610905
7	VGIVYK	0.6647688	23	KLVFFA	0.5582891
8	GGYLLG	0.6515245	24	QANKHI	0.5558876
9	AIIGLM	0.6325819	25	KHIIVA	0.5415985
10	LKVKVL	0.6315390	26	CTLWWG	0.5376764
11	VQIVGK	0.6104627	27	GAILSS	0.5367259
12	GVIGIA	0.6099401	28	NFGAIL	0.5251388
13	CTVWWG	0.6044970	29	VQIVKK	0.5182800
14	QGVCFR	0.6033099	30	LPTVYV	0.5177132
15	CQIVYK	0.5994211	31	VQICYK	0.5134984
16	VQCVYK	0.5896526	32	TCVTHR	0.5007680

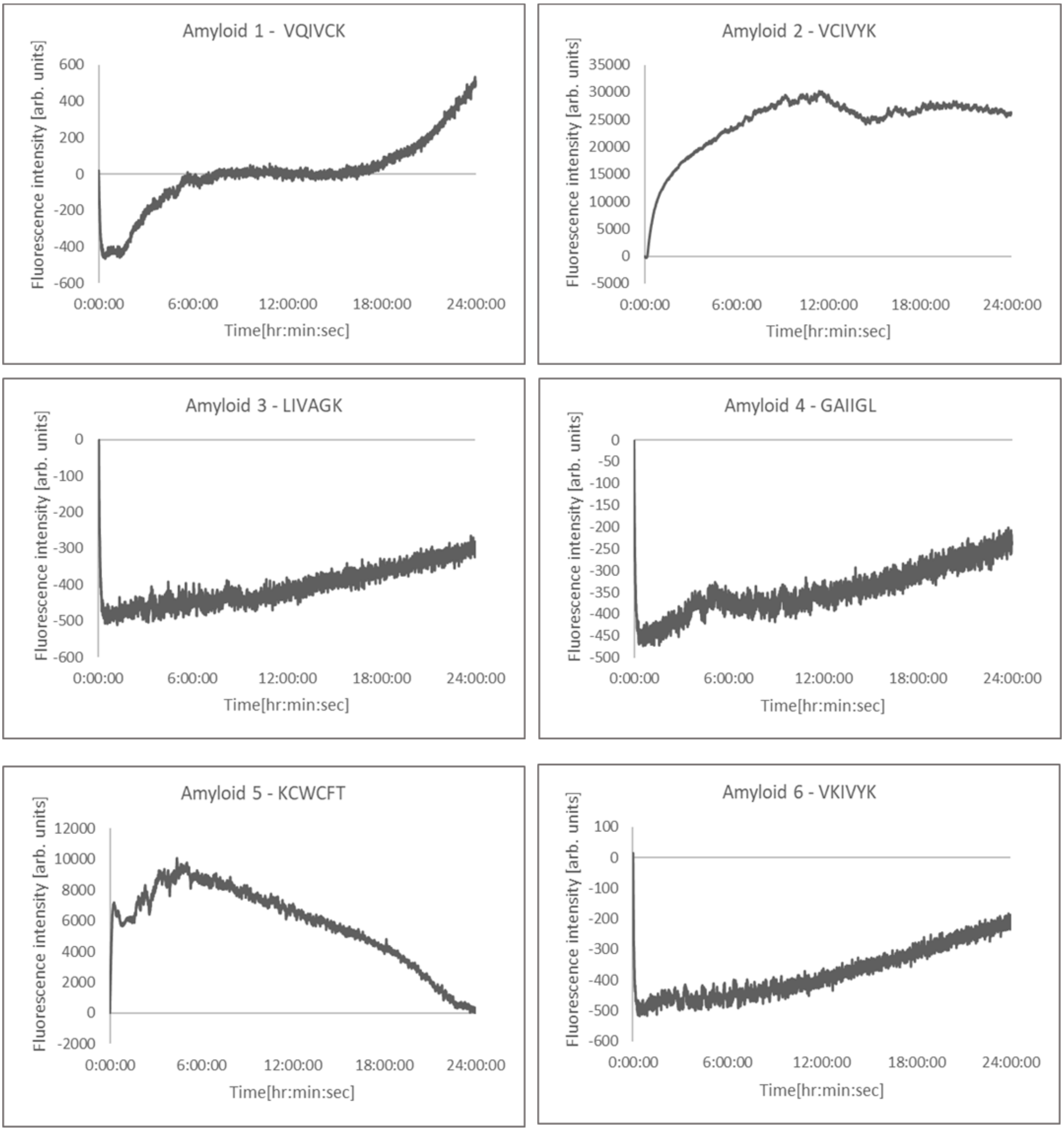

Figure S1. (See description on the next page.)**.**

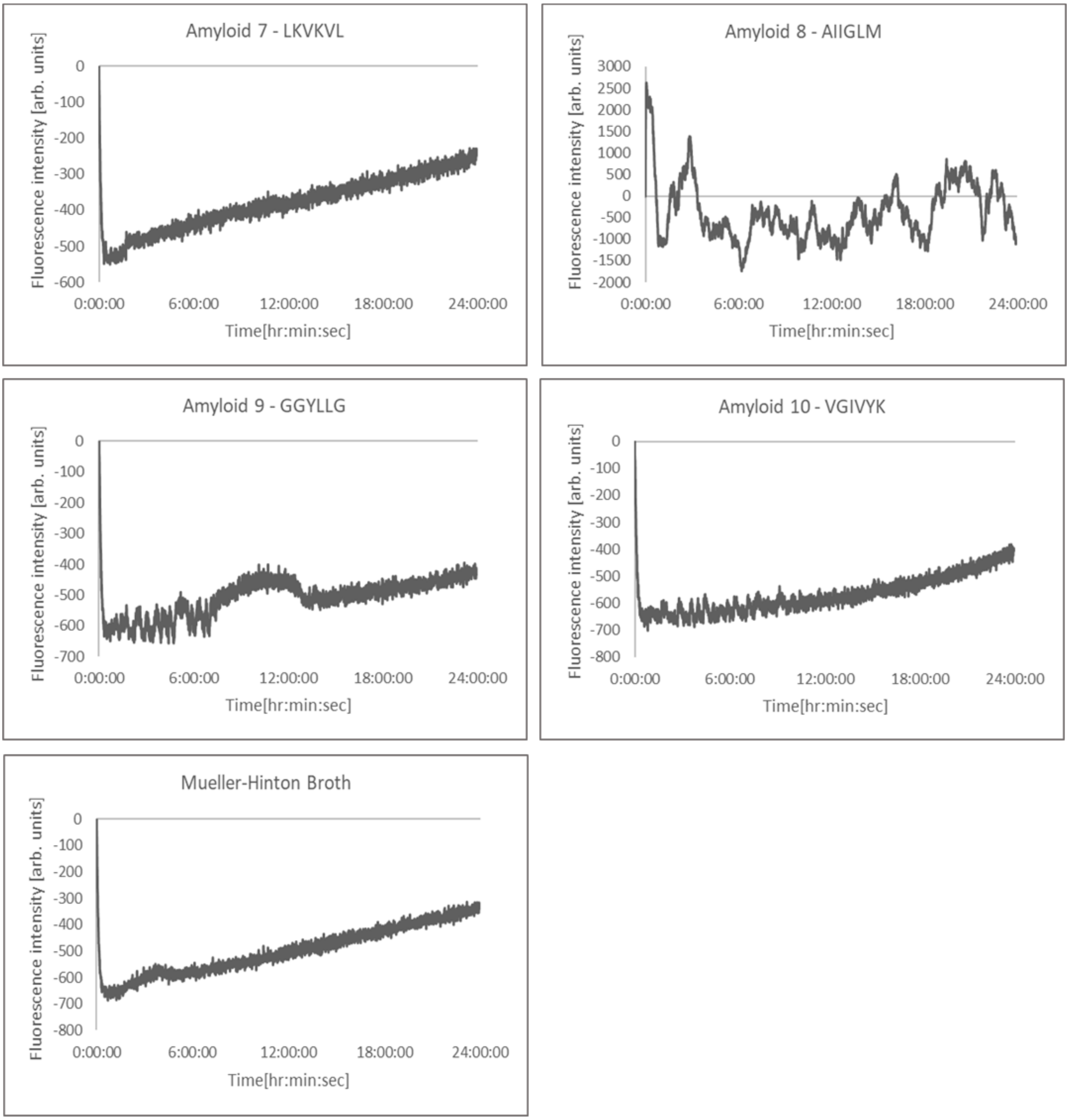

Figure S1. Experimental verification of the ten short amyloids to form aggregates in MHB medium with thioflavin T (λ_{ex} = 450 nm, λ_{em} = 490 nm). The x-axis represents time and the y-axis absorbance for the dye.

Table S2. Cytotoxic properties of short amyloids measured as dehydrogenase activity (MTT assay). Numbers from 128 to 0.25 represent dilutions of amyloids in µg/mL. The values in the table represent HEK-293 cell viability, i.e. the absorbance of the sample divided by the absorbance of the control, i.e. samples without a peptide.

Name	128	64	32	16	8	4	2	1	0,5	0,25
Amy 1	0,572	0,835	0,844	0,867	0,898	0,815	0,849	0,961	1,053	1,114
Amy 1	0,673	0,900	0,837	0,849	0,804	0,947	1,123	0,851	0,876	0,891
Amy 1	0,761	0,713	0,709	0,796	0,842	0,896	0,810	0,844	0,790	0,842
Amy 2	0,650	0,626	0,758	0,819	0,918	0,797	0,918	0,812	0,848	0,799
Amy 2	0,580	0,607	0,738	0,796	1,182	1,145	0,880	0,797	0,733	0,849
Amy 2	0,553	0,522	0,684	0,657	0,664	0,641	0,727	0,702	0,643	0,781
Amy 3	0,553	0,644	0,641	0,655	0,644	0,720	0,644	0,596	0,554	0,644
Amy 3	0,500	0,583	0,826	0,725	0,567	0,783	0,605	0,547	0,554	0,583
Amy 3	0,650	0,659	1,012	1,020	0,863	0,851	0,696	0,776	0,841	0,888
Amy 4	0,511	0,622	0,793	0,800	0,842	0,820	0,887	0,911	0,795	0,848
Amy 4	0,577	0,600	0,873	0,848	0,904	0,732	1,020	0,846	0,790	0,878
Amy 4	0,531	0,585	0,799	0,873	0,990	0,820	0,970	0,819	0,836	0,820
Amy 5	0,489	0,532	0,743	0,822	0,830	0,767	0,762	0,792	0,801	0,683
Amy 5	0,496	0,482	0,724	0,720	0,670	0,647	0,638	0,763	0,832	0,882
Amy 5	0,501	0,534	0,647	0,660	0,568	0,601	0,604	0,629	0,674	0,645
Amy 6	0,275	0,294	0,505	0,494	0,569	0,543	0,845	0,399	0,563	0,539
Amy 6	0,623	0,889	0,754	0,880	0,777	0,932	0,733	0,882	0,857	0,795
Amy 6	0,793	0,761	0,751	0,763	0,790	0,893	0,767	0,841	0,896	0,880
Amy 7	0,864	0,784	0,825	0,784	0,984	0,864	0,836	0,836	0,831	0,825
Amy 7	0,824	0,797	0,824	0,767	0,815	0,895	0,811	0,783	0,829	0,852
Amy 7	0,946	0,777	0,838	0,760	0,884	0,927	0,893	0,845	0,945	0,877
Amy 8	0,697	0,744	0,710	0,868	0,788	0,864	0,809	0,806	0,788	0,834
Amy 8	0,774	0,688	0,608	0,740	0,662	0,704	0,751	0,656	0,639	0,738
Amy 8	0,742	0,679	0,619	0,774	0,719	0,758	0,813	0,614	0,615	0,587
Amy 8	0,600	0,679	0,802	0,921	0,887	0,955	0,790	0,870	0,775	0,926
Amy 9	0,699	0,831	0,813	0,862	0,859	0,880	0,857	0,926	0,916	0,965
Amy 9	0,663	0,785	0,865	0,893	0,838	0,847	0,821	0,890	0,811	0,663
Amy 9	0,697	0,792	0,782	0,677	0,813	0,784	0,797	0,877	0,811	0,828
Amy 9	0,710	0,792	0,792	0,870	0,731	0,857	0,826	0,846	0,741	0,800
Amy 10	0,730	0,718	0,708	0,636	0,643	0,726	0,685	0,700	0,687	0,751
Amy 10	0,620	0,597	0,568	0,576	0,514	0,604	0,582	0,545	0,540	0,568
Amy 10	0,612	0,604	0,504	0,497	0,576	0,540	0,550	0,584	0,592	0,612

Table S3. Antimicrobial profiles determined by disc diffusion method for eight tested bacteria.

Antibiotic	Strain							
	**Gram-positive bacteria**				**Gram-negative bacteria**			
	S. aureus S16	*E. faecalis* 37VRE	*S. epidermidis* S22	*E. faecium* 2VRE	*K. pneumoniae* N111	*A. baumannii* 2800	*E. coli* 1471	*E. cloacae* 1476
Ampicillin		S		R				
Piperacyllin/tazobactam					R	R	R	R
Ampicillin/tazobactam					R			
Ampicillin/sulbactam							R	
Ticarcillin/clavulanic acid								
Amoxicillin/ clavulanic acid					R		R	
Cefoxitin	R		R					
Cefuroxime					R		R	
Cefotaxime					R		R	R
Ceftazidime					R	R	R	R
Cefepime					R	R	R	R
Aztreonam					R		R	
Doripenem					S		S	
Ertapenem							S	R
Imipenem		S		R	S	R	S	S
Meropenem					S	R	S	S
Ciprofloxacin	R		S		R	R	R	R
Levofloxacin						R		
Clindamycin	R		R					
Erythromycin	R		R					
Vancomycin	S	R	S	R				
Teicoplanin	S	S	S	S				
Streptomycin				R				
Gentamicin	S	R	S	R	R	R	R	
Amikacin					S	R	R	R
Tobramycin					R	R	R	R
Netylmycin	S		S					
Colistin					S	S		S
Linezolid	S							
Tetracycline			S					
Co-trimoxazole	S		R			R	S	R

S – susceptible; R – resistant; NT – not tested.

Table S4. Bacterial viability in the MHB medium with the addition of Amyloid 1 (VQIVCK). Numbers from 128 to 0.25 represent Amyloid 1 dilutions in µg/mL.

	**Amyloid 1 - VQIVCK**	**128**	**64**	**32**	**16**	**8**	**4**	**2**	**1**	**0.5**	**0.25**
**Gram +**	*S. aureus* S16	95%	99%	118%	114%	120%	123%	118%	118%	112%	108%
	E. faecalis 37VRE	49%	92%	106%	99%	112%	111%	109%	109%	99%	98%
	S. epidermidis S22	117%	104%	103%	98%	97%	96%	97%	99%	97%	97%
	E. faecium 2VRE	78%	101%	119%	108%	118%	124%	120%	121%	113%	111%
**Gram -**	*K. pneumoniae* N111	84%	83%	89%	84%	89%	93%	92%	95%	94%	96%
	A. baumannii 2800	118%	100%	96%	100%	98%	84%	98%	102%	98%	101%
	E. cloacae 1476	96%	97%	102%	98%	102%	98%	101%	101%	100%	99%
	E. coli 1471	89%	102%	102%	98%	98%	95%	97%	95%	96%	99%
**Ref.**	*E. coli* K12	106%	100%	99%	99%	100%	102%	100%	104%	98%	99%
	S. aureus ATCC 25923	98%	108%	108%	107%	105%	100%	103%	101%	98%	99%

Table S5. Bacterial viability in the MHB medium with the addition of Amyloid 2 (VCIVYK). Numbers from 128 to 0.25 represent Amyloid 2 dilutions in µg/mL.

	**Amyloid 2 - VCIVYK**	**128**	**64**	**32**	**16**	**8**	**4**	**2**	**1**	**0.5**	**0.25**
**Gram +**	*S. aureus* S16	115%	99%	100%	98%	105%	107%	108%	110%	104%	108%
	E. faecalis 37VRE	78%	83%	95%	85%	111%	116%	119%	116%	103%	94%
	S. epidermidis S22	91%	93%	93%	91%	90%	93%	92%	96%	93%	95%
	E. faecium 2VRE	63%	113%	137%	124%	148%	169%	140%	141%	115%	103%
**Gram -**	*K. pneumoniae* N111	92%	108%	117%	106%	106%	105%	103%	105%	101%	100%
	A. baumannii 2800	102%	97%	96%	93%	92%	96%	97%	95%	97%	94%
	E. cloacae 1476	101%	103%	104%	103%	104%	105%	102%	103%	102%	99%
	E. coli 1471	79%	101%	98%	93%	93%	88%	92%	88%	94%	92%
**Ref.**	*E. coli* K12	82%	93%	92%	93%	95%	98%	98%	99%	96%	99%
	S. aureus ATCC 25923	114%	109%	109%	109%	108%	104%	106%	101%	101%	98%

Table S6. Bacterial viability in the MHB medium with the addition of Amyloid 3 (LIVAGK). Numbers from 128 to 0.25 represent Amyloid 3 dilutions in µg/mL.

	**Amyloid 3 - LIVAGK**	**128**	**64**	**32**	**16**	**8**	**4**	**2**	**1**	**0.5**	**0.25**
**Gram +**	*S. aureus* S16	80%	112%	122%	110%	122%	120%	103%	105%	96%	86%
	E. faecalis 37VRE	76%	89%	82%	86%	85%	84%	85%	88%	86%	91%
	S. epidermidis S22	121%	97%	94%	91%	92%	92%	93%	95%	93%	93%
	E. faecium 2VRE	66%	83%	84%	86%	105%	108%	108%	116%	100%	99%
**Gram -**	*K. pneumoniae* N111	107%	110%	116%	110%	113%	114%	109%	109%	102%	104%
	A. baumannii 2800	109%	103%	92%	91%	96%	98%	98%	103%	95%	99%
	E. cloacae 1476	102%	105%	105%	103%	105%	105%	102%	101%	97%	98%
	E. coli 1471	83%	103%	102%	98%	97%	92%	93%	90%	94%	93%
**Ref.**	*E. coli* K12	111%	106%	104%	102%	104%	104%	103%	104%	101%	100%
	S. aureus ATCC 25923	109%	104%	105%	106%	103%	102%	107%	105%	105%	101%

Table S7. Bacterial viability in the MHB medium with the addition of Amyloid 4 (GAIIGL). Numbers from 128 to 0.25 represent Amyloid 4 dilutions in µg/mL.

	**Amyloid 4 - GAIIGL**	**128**	**64**	**32**	**16**	**8**	**4**	**2**	**1**	**0.5**	**0.25**
**Gram +**	*S. aureus* S16	118%	119%	125%	115%	124%	126%	117%	124%	98%	97%
	E. faecalis 37VRE	78%	93%	88%	92%	96%	98%	89%	96%	88%	92%
	S. epidermidis S22	119%	109%	106%	100%	102%	102%	95%	99%	98%	96%
	E. faecium 2VRE	84%	102%	112%	102%	119%	110%	102%	106%	98%	101%
**Gram -**	*K. pneumoniae* N111	85%	102%	106%	99%	102%	103%	104%	103%	101%	98%
	A. baumannii 2800	106%	103%	95%	102%	98%	99%	82%	99%	94%	99%
	E. cloacae 1476	91%	103%	101%	99%	99%	101%	100%	99%	98%	98%
	E. coli 1471	84%	111%	111%	107%	106%	104%	105%	104%	103%	104%
**Ref.**	*E. coli* K12	98%	101%	106%	104%	107%	111%	111%	112%	107%	104%
	S. aureus ATCC 25923	105%	113%	113%	109%	105%	105%	105%	102%	101%	96%

Table S8. Bacterial viability in the MHB medium with the addition of Amyloid 5 (KCWCFT). Numbers from 128 to 0.25 represent Amyloid 5 dilutions in µg/mL.

	**Amyloid 5 - KCWCFT**	**128**	**64**	**32**	**16**	**8**	**4**	**2**	**1**	**0.5**	**0.25**
**Gram +**	*S. aureus S16*	129%	87%	87%	87%	91%	103%	100%	113%	95%	97%
	E. faecalis 37VRE	128%	98%	84%	80%	82%	84%	84%	90%	86%	94%
	S. epidermidis S22	115%	91%	90%	91%	94%	78%	77%	79%	83%	88%
	E. faecium 2VRE	59%	64%	57%	69%	81%	95%	104%	116%	99%	110%
**Gram -**	*K. pneumoniae N111*	97%	100%	109%	104%	106%	102%	104%	104%	101%	101%
	A. baumannii 2800	189%	152%	135%	138%	104%	97%	86%	93%	86%	94%
	E. cloacae 1476	117%	108%	106%	106%	108%	107%	107%	106%	105%	105%
	E. coli 1471	76%	75%	82%	93%	101%	99%	100%	101%	98%	97%
**Ref.**	*E. coli K12*	97%	100%	84%	86%	81%	96%	97%	101%	100%	101%
	S. aureus ATCC 25923	122%	191%	150%	144%	84%	94%	105%	107%	110%	103%

Table S9. Bacterial viability in the MHB medium with the addition of Amyloid 6 (VKIVYK). Numbers from 128 to 0.25 represent Amyloid 6 dilutions in µg/mL.

	**Amyloid 6 - VKIVYK**	**128**	**64**	**32**	**16**	**8**	**4**	**2**	**1**	**0.5**	**0.25**
**Gram +**	*S. aureus S16*	103%	102%	119%	109%	116%	115%	110%	111%	108%	103%
	E. faecalis 37VRE	81%	83%	86%	95%	91%	99%	99%	96%	94%	91%
	S. epidermidis S22	110%	110%	106%	100%	94%	97%	92%	96%	100%	98%
	E. faecium 2VRE	50%	92%	117%	109%	125%	128%	128%	125%	118%	111%
**Gram -**	*K. pneumoniae N111*	80%	82%	93%	91%	98%	100%	99%	105%	103%	102%
	A. baumannii 2800	114%	82%	85%	77%	83%	86%	85%	89%	87%	86%
	E. cloacae 1476	87%	98%	100%	99%	103%	103%	101%	103%	100%	100%
	E. coli 1471	58%	98%	100%	97%	100%	98%	100%	100%	100%	99%
**Ref.**	*E. coli K12*	88%	95%	98%	94%	100%	102%	103%	105%	101%	100%
	S. aureus ATCC 25923	117%	111%	104%	104%	99%	99%	102%	99%	99%	96%

Table S10. Bacterial viability in the MHB medium with the addition of Amyloid 7 (LKVKVL). Numbers from 128 to 0.25 represent Amyloid 7 dilutions in µg/mL.

	**Amyloid 7 - LKVKVL**	**128**	**64**	**32**	**16**	**8**	**4**	**2**	**1**	**0.5**	**0.25**
**Gram +**	*S. aureus S16*	113%	121%	128%	118%	127%	128%	123%	110%	108%	105%
	E. faecalis 37VRE	85%	83%	98%	95%	99%	126%	109%	117%	116%	94%
	S. epidermidis S22	105%	96%	95%	95%	91%	92%	92%	97%	94%	96%
	E. faecium 2VRE	72%	103%	114%	101%	117%	121%	111%	117%	103%	109%
**Gram -**	*K. pneumoniae N111*	88%	102%	109%	102%	107%	106%	105%	105%	105%	103%
	A. baumannii 2800	122%	75%	71%	84%	79%	82%	80%	91%	92%	91%
	E. cloacae 1476	89%	98%	99%	98%	101%	100%	100%	102%	97%	97%
	E. coli 1471	62%	97%	98%	98%	102%	100%	101%	102%	100%	99%
**Ref.**	*E. coli K12*	88%	94%	96%	95%	99%	103%	101%	103%	100%	98%
	S. aureus ATCC 25923	108%	106%	100%	102%	103%	101%	104%	103%	101%	99%

Table S11. Bacterial viability in the MHB medium with the addition of Amyloid 8 (AIIGLM). Numbers from 128 to 0.25 represent Amyloid 8 dilutions in µg/mL.

	**Amyloid 8 - AIIGLM**	**128**	**64**	**32**	**16**	**8**	**4**	**2**	**1**	**0.5**	**0.25**
**Gram +**	*S. aureus S16*	101%	85%	103%	96%	103%	103%	101%	102%	97%	93%
	E. faecalis 37VRE	68%	68%	68%	75%	74%	88%	88%	90%	91%	92%
	S. epidermidis S22	114%	106%	106%	102%	93%	95%	87%	97%	93%	96%
	E. faecium 2VRE	63%	100%	125%	116%	125%	122%	121%	121%	110%	107%
**Gram -**	*K. pneumoniae N111*	76%	90%	101%	96%	102%	98%	101%	103%	102%	100%
	A. baumannii 2800	125%	91%	88%	92%	89%	90%	94%	89%	98%	86%
	E. cloacae 1476	91%	104%	103%	100%	101%	100%	98%	102%	98%	99%
	E. coli 1471	66%	99%	104%	101%	104%	102%	103%	102%	103%	101%
**Ref.**	*E. coli K12*	91%	91%	95%	93%	98%	99%	100%	102%	99%	99%
	S. aureus ATCC 25923	124%	110%	101%	100%	101%	104%	105%	105%	104%	101%

Table S12. Bacterial viability in the MHB medium with the addition of Amyloid 9 (GGYLLG). Numbers from 128 to 0.25 represent Amyloid 9 dilutions in µg/mL.

	**Amyloid 9 - GGYLLG**	**128**	**64**	**32**	**16**	**8**	**4**	**2**	**1**	**0.5**	**0.25**
**Gram +**	*S. aureus S16*	70%	106%	108%	98%	100%	95%	96%	103%	99%	99%
	E. faecalis 37VRE	32%	54%	38%	48%	53%	49%	53%	67%	67%	76%
	S. epidermidis S22	109%	96%	91%	92%	90%	91%	91%	96%	92%	96%
	E. faecium 2VRE	70%	99%	131%	115%	128%	138%	138%	139%	118%	116%
**Gram -**	*K. pneumoniae N111*	75%	101%	107%	105%	109%	107%	106%	108%	104%	105%
	A. baumannii 2800	112%	99%	91%	99%	97%	99%	97%	94%	92%	97%
	E. cloacae 1476	94%	96%	96%	99%	97%	98%	97%	100%	98%	97%
	E. coli 1471	77%	100%	103%	99%	101%	98%	98%	99%	100%	99%
**Ref.**	*E. coli K12*	94%	115%	118%	113%	116%	116%	113%	108%	108%	106%
	S. aureus ATCC 25923	109%	107%	101%	101%	102%	100%	102%	101%	99%	97%

Table S13. Bacterial viability in the MHB medium with the addition of Amyloid 10 (VGIVYK). Numbers from 128 to 0.25 represent Amyloid 10 dilutions in µg/mL.

	Amyloid 10 - VGIVYK	128	64	32	16	8	4	2	1	0.5	0.25
Gram +	*S. aureus S16*	78%	106%	107%	100%	105%	104%	106%	110%	103%	100%
	E. faecalis 37VRE	17%	38%	23%	40%	50%	57%	65%	82%	72%	77%
	S. epidermidis S22	120%	96%	93%	89%	90%	92%	91%	96%	91%	96%
	E. faecium 2VRE	66%	83%	97%	85%	95%	114%	119%	114%	97%	95%
Gram -	*K. pneumoniae N111*	90%	114%	117%	110%	110%	111%	107%	110%	106%	105%
	A. baumannii 2800	152%	110%	94%	95%	97%	92%	93%	96%	93%	100%
	E. cloacae 1476	103%	109%	109%	106%	111%	107%	104%	108%	105%	102%
	E. coli 1471	82%	103%	102%	99%	100%	97%	98%	97%	98%	97%
Ref.	*E. coli K12*	91%	106%	110%	107%	111%	117%	113%	114%	107%	104%
	S. aureus ATCC 25923	106%	96%	93%	95%	94%	94%	93%	94%	92%	91%

Table S14. Bacterial viability in the MHB medium with the addition of Colistin. Numbers from 128 to 0.25 represent Colistin dilutions in µg/mL.

	Colistin	128	64	32	16	8	4	2	1	0.5	0.25
Gram -	*K. pneumoniae* N111	2%	2%	1%	0%	0%	-2%	11%	73%	96%	101%
	A. baumannii 2800	7%	10%	5%	3%	1%	-1%	6%	38%	89%	97%
	E. cloacae 1476	4%	3%	2%	1%	12%	45%	71%	77%	87%	97%
	E. coli 1471	0%	0%	0%	-1%	-1%	2%	0%	87%	101%	101%
Ref.	*E. coli* K12	1%	-1%	-1%	-1%	1%	1%	6%	41%	87%	99%

Table S15. Bacterial viability in the MHB medium with the addition of Teicoplanin. Numbers from 128 to 0.25 represent Teicoplanin dilutions in µg/mL.

	Teicoplanin	128	64	32	16	8	4	2	1	0.5	0.25
Gram +	*S. aureus* S16	3%	3%	1%	0%	1%	0%	-2%	-1%	-2%	0%
	E. faecalis 37VRE	-3%	3%	2%	1%	0%	2%	-1%	0%	-7%	3%
	S. epidermidis S22	3%	2%	1%	0%	0%	1%	5%	41%	69%	85%
	E. faecium 2VRE	-4%	13%	5%	0%	-1%	4%	0%	6%	-3%	11%
Ref.	*S. aureus* ATCC 25923	3%	3%	-1%	-1%	0%	1%	-2%	0%	5%	68%

Table S16. Mean absorbance and standard deviation values for a given bacterial strain measured in the MHB medium with the addition of Amyloid 1 (VQIVCK). Numbers from 128 to 0.25 represent Amyloid 1 dilutions in µg/mL.

	**Amyloid 1 - VQIVCK**	**128**	**64**	**32**	**16**	**8**	**4**	**2**	**1**	**0.5**	**0.25**	**0**
**Gram +**	*S. aureus* S16	0.591±0.203	0.617±0.166	0.739±0.145	0.713±0.144	0.751±0.133	0.766±0.121	0.734±0.141	0.735±0.143	0.698±0.164	0.671±0.177	0.624±0.176
	E. faecalis 37VRE	0.156±0.052	0.291±0.131	0.337±0.134	0.315±0.123	0.355±0.112	0.354±0.128	0.346±0.124	0.347±0.127	0.316±0.113	0.311±0.132	0.318±0.145
	S. epidermidis S22	0.609±0.076	0.541±0.062	0.537±0.083	0.509±0.062	0.504±0.038	0.501±0.047	0.507±0.045	0.514±0.044	0.503±0.076	0.507±0.046	0.521±0.039
	E. faecium 2VRE	0.318±0.041	0.413±0.139	0.487±0.155	0.445±0.135	0.485±0.158	0.51±0.171	0.49±0.157	0.494±0.152	0.462±0.148	0.454±0.138	0.41±0.175
**Gram -**	*K. pneumoniae* N111	0.609±0.088	0.6±0.079	0.646±0.077	0.607±0.079	0.642±0.09	0.676±0.1	0.664±0.108	0.685±0.106	0.679±0.111	0.692±0.093	0.724±0.126
	A. baumannii 2800	0.685±0.072	0.581±0.125	0.561±0.121	0.583±0.101	0.569±0.09	0.488±0.226	0.572±0.102	0.59±0.091	0.569±0.078	0.589±0.079	0.581±0.101
	E. cloacae 1476	0.68±0.044	0.689±0.034	0.72±0.042	0.696±0.035	0.722±0.038	0.698±0.045	0.712±0.047	0.719±0.041	0.708±0.049	0.703±0.054	0.709±0.046
	E. coli 1471	0.758±0.172	0.868±0.129	0.868±0.145	0.829±0.122	0.835±0.133	0.805±0.135	0.825±0.131	0.807±0.147	0.815±0.146	0.837±0.148	0.848±0.173
**Ref.**	*E. coli* K12	0.515±0.029	0.486±0.028	0.483±0.026	0.48±0.029	0.487±0.03	0.497±0.044	0.485±0.038	0.504±0.058	0.476±0.039	0.478±0.039	0.485±0.032
	S. aureus ATCC 25923	0.39±0.062	0.428±0.073	0.428±0.08	0.425±0.079	0.417±0.069	0.395±0.058	0.409±0.058	0.401±0.049	0.387±0.044	0.392±0.052	0.396±0.065

Table S17. Mean absorbance and standard deviation values for a given bacterial strain measured in the MHB medium with the addition of Amyloid 2 (VCIVYK). Numbers from 128 to 0.25 represent Amyloid 2 dilutions in µg/mL.

	**Amyloid 2 - VCIVYK**	**128**	**64**	**32**	**16**	**8**	**4**	**2**	**1**	**0.5**	**0.25**	**0**
**Gram +**	*S. aureus S16*	0.53±0.066	0.459±0.106	0.463±0.126	0.454±0.123	0.483±0.11	0.496±0.122	0.498±0.124	0.507±0.11	0.482±0.099	0.498±0.101	0.462±0.069
	E. faecalis 37VRE	0.19±0.03	0.202±0.052	0.232±0.085	0.208±0.066	0.271±0.084	0.282±0.088	0.29±0.103	0.284±0.08	0.251±0.062	0.23±0.061	0.244±0.082
	S. epidermidis S22	0.33±0.035	0.338±0.034	0.336±0.067	0.331±0.043	0.325±0.028	0.337±0.037	0.332±0.025	0.348±0.032	0.335±0.028	0.344±0.03	0.362±0.026
	E. faecium 2VRE	0.129±0.032	0.233±0.143	0.283±0.178	0.257±0.142	0.306±0.165	0.349±0.168	0.29±0.178	0.291±0.159	0.237±0.126	0.212±0.092	0.207±0.085
**Gram -**	*K. pneumoniae N111*	0.664±0.135	0.773±0.116	0.837±0.104	0.761±0.099	0.764±0.138	0.754±0.112	0.736±0.106	0.751±0.106	0.722±0.089	0.715±0.094	0.718±0.122
	A. baumannii 2800	0.613±0.115	0.583±0.097	0.574±0.108	0.558±0.131	0.552±0.098	0.575±0.099	0.584±0.107	0.568±0.113	0.583±0.108	0.564±0.105	0.6±0.104
	E. cloacae 1476	0.657±0.049	0.673±0.042	0.677±0.042	0.668±0.034	0.677±0.042	0.684±0.045	0.665±0.046	0.672±0.045	0.663±0.05	0.646±0.051	0.651±0.058
	E. coli 1471	0.645±0.071	0.824±0.1	0.799±0.131	0.763±0.109	0.761±0.142	0.723±0.145	0.75±0.135	0.721±0.147	0.768±0.131	0.754±0.163	0.818±0.139
**Ref.**	*E. coli K12*	0.365±0.042	0.413±0.041	0.411±0.037	0.415±0.042	0.426±0.045	0.436±0.037	0.437±0.045	0.442±0.045	0.43±0.04	0.44±0.043	0.446±0.042
	S. aureus ATCC 25923	0.448±0.093	0.429±0.043	0.429±0.054	0.428±0.05	0.427±0.054	0.411±0.051	0.417±0.065	0.399±0.066	0.395±0.049	0.386±0.054	0.393±0.052

Table S18. Mean absorbance and standard deviation values for a given bacterial strain measured in the MHB medium with the addition of Amyloid 3 (LIVAGK). Numbers from 128 to 0.25 represent Amyloid 3 dilutions in µg/mL.

	**Amyloid 3 - LIVAGK**	**128**	**64**	**32**	**16**	**8**	**4**	**2**	**1**	**0.5**	**0.25**	**0**
Gram +	*S. aureus* S16	0.491±0.216	0.684±0.226	0.746±0.23	0.673±0.218	0.745±0.224	0.735±0.228	0.631±0.235	0.641±0.249	0.587±0.273	0.525±0.242	0.612±0.258
	E. faecalis 37VRE	0.123±0.019	0.144±0.021	0.133±0.018	0.138±0.019	0.138±0.023	0.136±0.026	0.137±0.023	0.142±0.027	0.139±0.021	0.147±0.019	0.162±0.023
	S. epidermidis S22	0.513±0.105	0.411±0.042	0.396±0.058	0.386±0.041	0.388±0.052	0.39±0.066	0.391±0.053	0.401±0.044	0.391±0.051	0.394±0.042	0.422±0.042
	E. faecium 2VRE	0.126±0.025	0.156±0.043	0.159±0.072	0.162±0.048	0.198±0.072	0.203±0.059	0.205±0.075	0.219±0.069	0.188±0.052	0.187±0.03	0.189±0.034
Gram -	*K. pneumoniae* N111	0.673±0.076	0.688±0.103	0.731±0.101	0.688±0.105	0.71±0.123	0.714±0.095	0.686±0.137	0.687±0.115	0.641±0.131	0.653±0.13	0.628±0.118
	A. baumannii 2800	0.604±0.088	0.569±0.144	0.509±0.135	0.503±0.123	0.531±0.138	0.539±0.137	0.54±0.106	0.566±0.111	0.526±0.085	0.548±0.118	0.552±0.099
	E. cloacae 1476	0.69±0.082	0.711±0.054	0.71±0.07	0.7±0.054	0.715±0.058	0.709±0.054	0.69±0.063	0.684±0.052	0.661±0.065	0.668±0.066	0.679±0.044
	E. coli 1471	0.726±0.136	0.904±0.12	0.892±0.121	0.854±0.105	0.844±0.124	0.806±0.117	0.81±0.113	0.787±0.136	0.823±0.138	0.809±0.145	0.874±0.112
Ref.	*E. coli* K12	0.53±0.041	0.505±0.06	0.497±0.035	0.49±0.042	0.499±0.034	0.495±0.026	0.494±0.039	0.497±0.037	0.485±0.045	0.479±0.038	0.478±0.04
	S. aureus ATCC 25923	0.5±0.085	0.479±0.054	0.483±0.049	0.487±0.054	0.472±0.041	0.468±0.051	0.491±0.034	0.482±0.031	0.482±0.036	0.467±0.032	0.46±0.045

Table S19. Mean absorbance and standard deviation values for a given bacterial strain measured in the MHB medium with the addition of Amyloid 4 (GAIIGL). Numbers from 128 to 0.25 represent Amyloid 4 dilutions in µg/mL.

	**Amyloid 4 - GAIIGL**	**128**	**64**	**32**	**16**	**8**	**4**	**2**	**1**	**0.5**	**0.25**	**0**
Gram +	*S. aureus* S16	0.544±0.241	0.551±0.224	0.58±0.227	0.531±0.198	0.574±0.199	0.583±0.201	0.543±0.222	0.575±0.175	0.451±0.183	0.45±0.121	0.463±0.151
	E. faecalis 37VRE	0.119±0.027	0.142±0.022	0.135±0.02	0.141±0.019	0.146±0.019	0.149±0.037	0.136±0.018	0.147±0.015	0.134±0.019	0.141±0.018	0.153±0.022
	S. epidermidis S22	0.508±0.076	0.464±0.112	0.452±0.106	0.426±0.089	0.437±0.09	0.435±0.088	0.405±0.055	0.424±0.068	0.42±0.069	0.412±0.052	0.427±0.039
	E. faecium 2VRE	0.153±0.129	0.185±0.057	0.203±0.07	0.186±0.043	0.217±0.066	0.201±0.068	0.185±0.051	0.193±0.046	0.179±0.025	0.184±0.031	0.182±0.034
Gram -	*K. pneumoniae* N111	0.621±0.056	0.742±0.107	0.769±0.117	0.725±0.112	0.742±0.128	0.752±0.137	0.759±0.128	0.751±0.134	0.733±0.123	0.717±0.118	0.728±0.131
	A. baumannii 2800	0.608±0.093	0.59±0.06	0.546±0.102	0.582±0.071	0.562±0.079	0.566±0.092	0.468±0.241	0.568±0.094	0.538±0.102	0.565±0.096	0.573±0.111
	E. cloacae 1476	0.584±0.071	0.659±0.051	0.643±0.04	0.635±0.04	0.634±0.053	0.646±0.048	0.637±0.056	0.632±0.049	0.626±0.068	0.625±0.067	0.639±0.058
	E. coli 1471	0.651±0.122	0.866±0.109	0.863±0.118	0.831±0.102	0.823±0.12	0.811±0.125	0.816±0.134	0.809±0.15	0.8±0.134	0.813±0.13	0.778±0.152
Ref.	*E. coli* K12	0.459±0.045	0.473±0.079	0.497±0.111	0.488±0.093	0.504±0.104	0.52±0.11	0.522±0.118	0.527±0.109	0.501±0.091	0.49±0.069	0.47±0.033
	S. aureus ATCC 25923	0.528±0.084	0.567±0.051	0.569±0.063	0.549±0.065	0.527±0.056	0.525±0.057	0.53±0.07	0.51±0.074	0.506±0.067	0.483±0.059	0.503±0.066

Table S20. Mean absorbance and standard deviation values for a given bacterial strain measured in the MHB medium with the addition of Amyloid 5 (KCWCFT). Numbers from 128 to 0.25 represent Amyloid 5 dilutions in µg/mL.

	**Amyloid 5 - KCWCFT**	**128**	**64**	**32**	**16**	**8**	**4**	**2**	**1**	**0.5**	**0.25**	**0**
**Gram +**	*S. aureus* S16	0.717±0.127	0.483±0.11	0.485±0.144	0.483±0.154	0.508±0.18	0.571±0.224	0.554±0.217	0.627±0.205	0.527±0.226	0.54±0.222	0.556±0.226
	E. faecalis 37VRE	0.204±0.092	0.156±0.035	0.134±0.025	0.128±0.032	0.131±0.03	0.135±0.032	0.134±0.031	0.144±0.031	0.138±0.04	0.15±0.038	0.16±0.036
	S. epidermidis S22	0.581±0.129	0.459±0.069	0.456±0.045	0.461±0.121	0.474±0.142	0.397±0.067	0.387±0.045	0.4±0.043	0.419±0.075	0.447±0.123	0.506±0.129
	E. faecium 2VRE	0.113±0.029	0.122±0.019	0.11±0.015	0.132±0.026	0.155±0.038	0.183±0.038	0.2±0.059	0.222±0.063	0.191±0.03	0.21±0.047	0.192±0.028
**Gram -**	*K. pneumoniae* N111	0.708±0.09	0.727±0.082	0.794±0.119	0.758±0.102	0.769±0.128	0.74±0.153	0.756±0.132	0.759±0.136	0.734±0.139	0.738±0.13	0.728±0.155
	A. baumannii 2800	0.789±0.057	0.635±0.217	0.565±0.215	0.578±0.177	0.435±0.165	0.404±0.14	0.36±0.097	0.39±0.104	0.358±0.108	0.393±0.119	0.418±0.114
	E. cloacae 1476	0.768±0.077	0.705±0.058	0.694±0.048	0.696±0.046	0.707±0.059	0.703±0.056	0.699±0.064	0.692±0.067	0.687±0.065	0.685±0.048	0.655±0.063
	E. coli 1471	0.686±0.107	0.678±0.101	0.738±0.1	0.838±0.155	0.913±0.153	0.893±0.135	0.907±0.151	0.909±0.121	0.889±0.129	0.879±0.151	0.903±0.173
**Ref.**	*E. coli* K12	0.549±0.05	0.566±0.087	0.474±0.071	0.489±0.053	0.458±0.054	0.547±0.158	0.549±0.158	0.572±0.169	0.566±0.179	0.571±0.163	0.567±0.173
	S. aureus ATCC 25923	0.527±0.082	0.823±0.156	0.646±0.112	0.619±0.132	0.361±0.059	0.404±0.078	0.453±0.108	0.46±0.098	0.475±0.105	0.445±0.086	0.431±0.106

Table S21. Mean absorbance and standard deviation values for a given bacterial strain measured in the MHB medium with the addition of Amyloid 6 (VKIVYK). Numbers from 128 to 0.25 represent Amyloid 6 dilutions in µg/mL.

	**Amyloid 6 - VKIVYK**	**128**	**64**	**32**	**16**	**8**	**4**	**2**	**1**	**0.5**	**0.25**	**0**
**Gram +**	*S. aureus* S16	0.544±0.172	0.541±0.171	0.629±0.156	0.576±0.132	0.615±0.165	0.611±0.156	0.584±0.15	0.589±0.17	0.574±0.153	0.545±0.118	0.53±0.106
	E. faecalis 37VRE	0.121±0.036	0.122±0.037	0.128±0.051	0.141±0.061	0.135±0.036	0.146±0.056	0.146±0.063	0.143±0.041	0.14±0.052	0.135±0.035	0.148±0.039
	S. epidermidis S22	0.439±0.113	0.442±0.112	0.424±0.096	0.401±0.078	0.377±0.076	0.388±0.068	0.367±0.058	0.385±0.048	0.401±0.072	0.394±0.059	0.4±0.044
	E. faecium 2VRE	0.178±0.018	0.328±0.186	0.42±0.211	0.389±0.197	0.445±0.186	0.459±0.183	0.458±0.189	0.445±0.201	0.423±0.178	0.397±0.198	0.358±0.165
**Gram -**	*K. pneumoniae* N111	0.572±0.043	0.59±0.077	0.668±0.114	0.653±0.106	0.705±0.125	0.719±0.122	0.713±0.13	0.752±0.134	0.736±0.13	0.731±0.136	0.717±0.146
	A. baumannii 2800	0.544±0.11	0.393±0.135	0.409±0.119	0.37±0.085	0.399±0.103	0.41±0.133	0.407±0.146	0.428±0.111	0.417±0.139	0.412±0.133	0.479±0.152
	E. cloacae 1476	0.607±0.06	0.688±0.043	0.703±0.051	0.693±0.034	0.722±0.054	0.724±0.051	0.707±0.053	0.723±0.049	0.702±0.059	0.699±0.06	0.701±0.058
	E. coli 1471	0.539±0.039	0.918±0.189	0.931±0.195	0.91±0.18	0.934±0.178	0.919±0.178	0.932±0.178	0.932±0.176	0.934±0.183	0.929±0.174	0.934±0.186
**Ref.**	*E. coli* K12	0.595±0.064	0.644±0.094	0.665±0.091	0.637±0.096	0.678±0.091	0.689±0.091	0.701±0.103	0.709±0.108	0.688±0.101	0.676±0.099	0.678±0.119
	S. aureus ATCC 25923	0.468±0.082	0.446±0.108	0.418±0.106	0.417±0.096	0.398±0.114	0.396±0.1	0.408±0.11	0.398±0.102	0.396±0.1	0.384±0.091	0.401±0.097

Table S22. Mean absorbance and standard deviation values for a given bacterial strain measured in the MHB medium with the addition of Amyloid 7 (LKVKVL). Numbers from 128 to 0.25 represent Amyloid 7 dilutions in µg/mL.

	**Amyloid 7 - LKVKVL**	**128**	**64**	**32**	**16**	**8**	**4**	**2**	**1**	**0.5**	**0.25**	**0**
**Gram +**	*S. aureus* S16	0.511±0.147	0.543±0.122	0.575±0.143	0.53±0.149	0.572±0.138	0.577±0.135	0.551±0.12	0.493±0.109	0.486±0.104	0.474±0.101	0.45±0.094
	E. faecalis 37VRE	0.141±0.051	0.138±0.033	0.163±0.063	0.158±0.058	0.165±0.065	0.209±0.111	0.181±0.073	0.195±0.071	0.193±0.073	0.156±0.033	0.166±0.061
	S. epidermidis S22	0.403±0.049	0.368±0.036	0.366±0.069	0.366±0.053	0.351±0.024	0.356±0.03	0.353±0.026	0.373±0.031	0.362±0.033	0.369±0.033	0.385±0.032
	E. faecium 2VRE	0.149±0.025	0.215±0.118	0.236±0.111	0.211±0.097	0.244±0.097	0.252±0.111	0.231±0.096	0.242±0.108	0.214±0.071	0.226±0.076	0.208±0.054
**Gram -**	*K. pneumoniae* N111	0.633±0.062	0.736±0.139	0.781±0.143	0.736±0.113	0.768±0.141	0.763±0.128	0.755±0.137	0.754±0.149	0.755±0.131	0.743±0.118	0.719±0.139
	A. baumannii 2800	0.587±0.148	0.363±0.145	0.341±0.138	0.404±0.148	0.38±0.109	0.393±0.141	0.384±0.104	0.436±0.104	0.443±0.12	0.436±0.104	0.481±0.114
	E. cloacae 1476	0.63±0.04	0.691±0.042	0.7±0.054	0.694±0.051	0.712±0.055	0.703±0.052	0.707±0.052	0.718±0.052	0.682±0.05	0.685±0.052	0.707±0.057
	E. coli 1471	0.559±0.068	0.872±0.197	0.878±0.202	0.877±0.181	0.914±0.145	0.899±0.169	0.909±0.169	0.913±0.157	0.895±0.195	0.888±0.201	0.897±0.218
**Ref.**	*E. coli* K12	0.648±0.079	0.689±0.09	0.704±0.092	0.693±0.093	0.726±0.094	0.756±0.085	0.743±0.089	0.754±0.091	0.732±0.099	0.719±0.096	0.732±0.112
	S. aureus ATCC 25923	0.537±0.07	0.529±0.077	0.501±0.091	0.511±0.082	0.512±0.087	0.504±0.092	0.518±0.096	0.516±0.084	0.503±0.078	0.494±0.077	0.499±0.079

Table S23. Mean absorbance and standard deviation values for a given bacterial strain measured in the MHB medium with the addition of Amyloid 8 (AIIGLM). Numbers from 128 to 0.25 represent Amyloid 8 dilutions in µg/mL.

	**Amyloid 8 - AIIGLM**	**128**	**64**	**32**	**16**	**8**	**4**	**2**	**1**	**0.5**	**0.25**	**0**
**Gram +**	*S. aureus* S16	0.582±0.115	0.491±0.118	0.594±0.169	0.554±0.131	0.595±0.174	0.593±0.16	0.585±0.148	0.592±0.153	0.559±0.125	0.539±0.129	0.578±0.135
	E. faecalis 37VRE	0.127±0.036	0.127±0.024	0.127±0.033	0.139±0.033	0.139±0.028	0.165±0.073	0.164±0.055	0.169±0.049	0.17±0.064	0.172±0.076	0.187±0.057
	S. epidermidis S22	0.464±0.089	0.431±0.121	0.433±0.115	0.414±0.113	0.377±0.072	0.385±0.059	0.353±0.044	0.396±0.056	0.38±0.08	0.392±0.071	0.407±0.077
	E. faecium 2VRE	0.182±0.045	0.287±0.123	0.358±0.147	0.334±0.114	0.36±0.151	0.35±0.15	0.347±0.131	0.348±0.157	0.315±0.163	0.308±0.135	0.287±0.112
**Gram -**	*K. pneumoniae* N111	0.619±0.1	0.733±0.153	0.819±0.126	0.785±0.136	0.827±0.15	0.801±0.14	0.819±0.142	0.836±0.125	0.833±0.113	0.814±0.149	0.814±0.166
	A. baumannii 2800	0.607±0.112	0.442±0.124	0.429±0.122	0.446±0.135	0.432±0.113	0.438±0.134	0.458±0.112	0.434±0.087	0.475±0.191	0.417±0.087	0.487±0.127
	E. cloacae 1476	0.608±0.055	0.693±0.046	0.69±0.057	0.671±0.049	0.672±0.048	0.671±0.052	0.655±0.045	0.679±0.048	0.658±0.052	0.66±0.038	0.668±0.049
	E. coli 1471	0.601±0.089	0.909±0.201	0.952±0.189	0.928±0.182	0.954±0.156	0.932±0.146	0.94±0.152	0.934±0.142	0.941±0.153	0.928±0.152	0.916±0.197
**Ref.**	*E. coli* K12	0.648±0.083	0.654±0.087	0.682±0.099	0.662±0.095	0.701±0.105	0.712±0.111	0.719±0.12	0.733±0.124	0.711±0.134	0.707±0.145	0.716±0.174
	S. aureus ATCC 25923	0.571±0.067	0.507±0.09	0.466±0.089	0.462±0.094	0.466±0.079	0.48±0.09	0.483±0.07	0.484±0.084	0.478±0.072	0.466±0.075	0.46±0.069

Table S24. Mean absorbance and standard deviation values for a given bacterial strain measured in the MHB medium with the addition of Amyloid 9 (GGYLLG). Numbers from 128 to 0.25 represent Amyloid 9 dilutions in μg/mL.

	**Amyloid 9 - GGYLLG**	**128**	**64**	**32**	**16**	**8**	**4**	**2**	**1**	**0.5**	**0.25**	**0**
**Gram +**	*S. aureus* S16	0.469±0.112	0.708±0.178	0.722±0.184	0.659±0.186	0.672±0.189	0.635±0.214	0.645±0.207	0.69±0.205	0.664±0.186	0.662±0.19	0.671±0.208
	E. faecalis 37VRE	0.022±0.024	0.037±0.032	0.026±0.046	0.033±0.03	0.037±0.034	0.034±0.039	0.036±0.028	0.047±0.025	0.046±0.028	0.053±0.029	0.069±0.03
	S. epidermidis S22	0.412±0.079	0.364±0.057	0.344±0.048	0.348±0.047	0.339±0.034	0.343±0.045	0.343±0.037	0.361±0.04	0.348±0.041	0.363±0.034	0.378±0.035
	E. faecium 2VRE	0.153±0.019	0.217±0.084	0.288±0.129	0.253±0.114	0.28±0.136	0.302±0.114	0.303±0.139	0.305±0.129	0.259±0.146	0.255±0.138	0.22±0.117
**Gram -**	*K. pneumoniae* N111	0.573±0.051	0.772±0.151	0.823±0.149	0.805±0.165	0.836±0.139	0.825±0.152	0.815±0.164	0.827±0.135	0.798±0.12	0.808±0.136	0.768±0.149
	A. baumannii 2800	0.577±0.126	0.509±0.183	0.47±0.123	0.507±0.124	0.497±0.103	0.509±0.117	0.497±0.091	0.483±0.116	0.475±0.117	0.496±0.09	0.514±0.085
	E. cloacae 1476	0.641±0.079	0.658±0.069	0.654±0.067	0.677±0.071	0.662±0.072	0.67±0.065	0.662±0.06	0.683±0.06	0.671±0.071	0.663±0.07	0.683±0.076
	E. coli 1471	0.727±0.098	0.942±0.134	0.97±0.083	0.93±0.074	0.952±0.079	0.919±0.077	0.926±0.083	0.931±0.077	0.941±0.087	0.935±0.08	0.941±0.111
**Ref.**	*E. coli* K12	0.596±0.119	0.73±0.139	0.747±0.129	0.715±0.111	0.738±0.11	0.735±0.107	0.716±0.116	0.685±0.2	0.686±0.094	0.67±0.092	0.635±0.093
	S. aureus ATCC 25923	0.51±0.088	0.501±0.078	0.471±0.077	0.472±0.076	0.478±0.084	0.468±0.066	0.478±0.084	0.473±0.079	0.463±0.086	0.456±0.078	0.468±0.093

Table S25. Mean absorbance and standard deviation values for a given bacterial strain measured in the MHB medium with the addition of Amyloid 10 (VGIVYK). Numbers from 128 to 0.25 represent Amyloid 10 dilutions in μg/mL.

	**Amyloid 10 - VGIVYK**	**128**	**64**	**32**	**16**	**8**	**4**	**2**	**1**	**0.5**	**0.25**	**0**
**Gram +**	*S. aureus* S16	0.477±0.16	0.646±0.164	0.654±0.184	0.612±0.177	0.639±0.183	0.634±0.187	0.646±0.193	0.67±0.203	0.627±0.195	0.613±0.188	0.61±0.182
	E. faecalis 37VRE	0.011±0.026	0.024±0.037	0.014±0.043	0.025±0.038	0.032±0.046	0.036±0.035	0.041±0.044	0.052±0.036	0.046±0.038	0.049±0.033	0.063±0.035
	S. epidermidis S22	0.452±0.132	0.364±0.065	0.351±0.053	0.335±0.049	0.341±0.045	0.349±0.051	0.343±0.048	0.362±0.042	0.345±0.045	0.363±0.041	0.378±0.039
	E. faecium 2VRE	0.16±0.057	0.2±0.098	0.235±0.116	0.206±0.101	0.231±0.111	0.275±0.138	0.288±0.136	0.276±0.13	0.235±0.111	0.232±0.109	0.243±0.094
**Gram -**	*K. pneumoniae* N111	0.558±0.053	0.704±0.098	0.722±0.115	0.683±0.102	0.684±0.118	0.685±0.092	0.664±0.13	0.681±0.106	0.659±0.12	0.649±0.12	0.619±0.145
	A. baumannii 2800	0.501±0.092	0.361±0.124	0.31±0.087	0.313±0.093	0.321±0.087	0.302±0.096	0.307±0.099	0.315±0.071	0.307±0.086	0.328±0.105	0.329±0.089
	E. cloacae 1476	0.656±0.037	0.696±0.058	0.698±0.06	0.681±0.045	0.707±0.05	0.683±0.045	0.664±0.053	0.688±0.049	0.669±0.061	0.655±0.052	0.639±0.06
	E. coli 1471	0.779±0.14	0.973±0.059	0.968±0.055	0.933±0.038	0.946±0.044	0.916±0.04	0.923±0.04	0.921±0.05	0.931±0.052	0.918±0.079	0.946±0.075
**Ref.**	*E. coli* K12	0.549±0.039	0.639±0.12	0.66±0.129	0.641±0.128	0.667±0.144	0.703±0.141	0.68±0.147	0.685±0.14	0.641±0.133	0.622±0.126	0.601±0.116
	S. aureus ATCC 25923	0.456±0.083	0.413±0.052	0.403±0.05	0.411±0.041	0.407±0.052	0.406±0.055	0.403±0.055	0.404±0.043	0.396±0.059	0.393±0.055	0.432±0.074

Table S26. Mean absorbance and standard deviation values for a given bacterial strain measured in the MHB medium with the addition of Colistin. Numbers from 128 to 0.25 represent colistin dilutions in µg/mL.

	**Colistin**	**128**	**64**	**32**	**16**	**8**	**4**	**2**	**1**	**0.5**	**0.25**	**0**
**Gram -**	*K. pneumoniae* N111	0.017±0.007	0.017±0.01	0.007±0.007	0±0.008	0±0.009	-0.012±0.017	0.088±0.165	0.561±0.135	0.74±0.083	0.783±0.045	0.772±0.047
	A. baumannii 2800	0.025±0.03	0.033±0.035	0.018±0.03	0.011±0.028	0.004±0.014	-0.002±0.017	0.02±0.01	0.129±0.017	0.3±0.044	0.326±0.057	0.338±0.047
	E. cloacae 1476	0.032±0.018	0.023±0.009	0.018±0.01	0.011±0.009	0.091±0.13	0.357±0.228	0.56±0.05	0.602±0.053	0.687±0.069	0.762±0.082	0.787±0.115
	E. coli 1471	0.002±0.012	-0.002±0.011	0.001±0.012	-0.005±0.005	-0.007±0.008	0.021±0.091	-0.001±0.009	0.724±0.07	0.84±0.078	0.842±0.085	0.83±0.088
**Ref.**	*E. coli* K12	0.006±0.011	-0.008±0.007	-0.004±0.006	-0.008±0.013	0.003±0.054	0.004±0.063	0.032±0.081	0.231±0.207	0.496±0.102	0.563±0.058	0.569±0.041

Table S27. Mean absorbance and standard deviation values for a given bacterial strain measured in the MHB medium with the addition of Teicoplanin. Numbers from 128 to 0.25 represent teicoplanin dilutions in µg/mL.

	**Teicoplanin**	**128**	**64**	**32**	**16**	**8**	**4**	**2**	**1**	**0.5**	**0.25**	**0**
**Gram +**	*S. aureus* S16	0.016±0.014	0.02±0.013	0.008±0.007	-0.001±0.006	0.004±0.01	0±0.009	-0.01±0.011	-0.004±0.014	-0.012±0.01	0.003±0.017	0.608±0.14
	E. faecalis 37VRE	-0.01±0.008	0.01±0.013	0.006±0.008	0.002±0.016	0±0.014	0.006±0.009	-0.003±0.011	0±0.008	-0.021±0.007	0.01±0.012	0.31±0.094
	S. epidermidis S22	0.02±0.009	0.018±0.02	0.011±0.012	-0.002±0.006	0±0.009	0.004±0.005	0.039±0.052	0.314±0.081	0.521±0.077	0.649±0.099	0.76±0.147
	E. faecium 2VRE	-0.009±0.012	0.028±0.016	0.01±0.017	0±0.009	-0.003±0.01	0.009±0.008	0±0.006	0.012±0.006	-0.006±0.006	0.024±0.007	0.214±0.175
**Ref.**	*S. aureus* ATCC 25923	0.012±0.01	0.013±0.01	-0.005±0.009	-0.004±0.007	0±0.008	0.005±0.004	-0.007±0.012	0±0.013	0.019±0.047	0.267±0.089	0.393±0.175

Table S28. Parameters of regression analyses for viability bacteria subjected to amyloids. The table includes cases for which the proportion of the explained variation was greater than 0.25 and the viability of bacteria was statistically significantly negatively correlated with the peptide concentration. The 'Amy' states for 'Amyloid' and R^2 for coefficient of determination.

Amyloid	Species	R^2	p-value	slope	Amyloid	Species	R^2	p-value	slope
Amy 1	*E. faecalis* 37VRE	0.645	3.8E-17	-0.395	Amy 6	*E. cloacae* 1476	0.400	1.3E-12	-0.104
Amy 1	*E. faecium* 2VRE	0.381	1.0E-04	-0.460	Amy 6	*E. coli* 1471	0.500	2.9E-17	-0.282
Amy 1	*K. pneumoniae* N111	0.384	1.5E-04	-0.083	Amy 6	*E. coli* K12	0.509	1.9E-07	-0.107
Amy 1	*S. aureus* S16	0.357	1.5E-04	-0.168	Amy 6	*E. faecium* 2VRE	0.465	4.9E-11	-0.504
Amy 10	*E. coli* 1471	0.266	2.4E-04	-0.070	Amy 6	*K. pneumoniae* N111	0.375	3.0E-12	-0.187
Amy 10	*E. faecalis* 37VRE	0.598	4.0E-05	-0.465	Amy 7	*E. cloacae* 1476	0.395	4.5E-09	-0.068
Amy 10	*E. faecium* 2VRE	0.288	1.5E-04	-0.305	Amy 7	*E. coli* 1471	0.389	2.5E-11	-0.261
Amy 10	*K. pneumoniae* N111	0.378	2.3E-03	-0.104	Amy 7	*E. coli* K12	0.468	1.0E-08	-0.098
Amy 10	*S. aureus* S16	0.401	5.3E-04	-0.156	Amy 7	*E. faecalis* 37VRE	0.308	8.7E-03	-0.215
Amy 2	*E. coli* 1471	0.623	8.5E-03	-0.056	Amy 7	*E. faecium* 2VRE	0.316	7.0E-04	-0.276
Amy 2	*E. coli* K12	0.940	6.7E-06	-0.121	Amy 7	*K. pneumoniae* N111	0.353	1.5E-04	-0.112
Amy 2	*E. faecalis* 37VRE	0.554	4.1E-07	-0.247	Amy 8	*E. coli* 1471	0.362	2.0E-14	-0.242
Amy 2	*E. faecium* 2VRE	0.516	1.4E-06	-0.509	Amy 8	*E. coli* K12	0.777	4.1E-07	-0.077
Amy 3	*E. faecalis* 37VRE	0.624	6.8E-03	-0.070	Amy 8	*E. faecalis* 37VRE	0.384	5.6E-05	-0.182
Amy 3	*E. faecium* 2VRE	0.419	9.3E-08	-0.299	Amy 8	*E. faecium* 2VRE	0.446	1.2E-04	-0.322
Amy 4	*E. cloacae* 1476	0.265	4.8E-03	-0.040	Amy 8	*K. pneumoniae* N111	0.355	1.8E-12	-0.189
Amy 4	*E. coli* 1471	0.313	2.0E-05	-0.111	Amy 9	*E. cloacae* 1476	0.401	2.0E-02	-0.037
Amy 4	*E. coli* K12	0.468	2.9E-03	-0.088	Amy 9	*E. coli* 1471	0.437	1.0E-14	-0.140
Amy 4	*E. faecalis* 37VRE	0.259	5.2E-03	-0.106	Amy 9	*E. coli* K12	0.600	7.0E-04	-0.094
Amy 4	*E. faecium* 2VRE	0.253	3.4E-02	-0.135	Amy 9	*E. faecalis* 37VRE	0.641	4.9E-03	-0.232
Amy 4	*K. pneumoniae* N111	0.410	1.6E-04	-0.102	Amy 9	*E. faecium* 2VRE	0.457	6.3E-08	-0.440
Amy 5	*E. coli* 1471	0.432	1.8E-17	-0.222	Amy 9	*K. pneumoniae* N111	0.436	4.5E-12	-0.221
Amy 5	*E. faecium* 2VRE	0.425	1.0E-14	-0.391	Amy 9	*S. aureus* S16	0.323	1.3E-03	-0.153

Table S29. Experimentally confirmed non-AMPs.

UniProt entry	Protein name	Organism	Length
O93454	Plasticin-DA1	*Agalychnis dacnicolor*	71
Q8ISL8	Apisimin	*Apis mellifera*	78
E4Z7G0	Kassorin-M	*Phlyctimantis maculatus*	65
C0HJV7	Met-lysine-1a	*Lachesana tarabaevi*	121
C0HJV8	Met-lysine-1b	*Lachesana tarabaevi*	121
A0A1W6EVN2	Ampulexin 2	*Ampulex compressa*	50
A0A1W6EVM7	Ampulexin 1	*Ampulex compressa*	50
C0HLE0	Plasticin-TR	*Phyllomedusa trinitatis*	22
P85507	Ranaspumin	*Leptodactylus vastus*	217
P84270	Dahlein-5.4	*Ranoidea dahlii*	21
P84267	Dahlein-5.1	*Ranoidea dahlii*	20
P84272	Dahlein-5.6	*Ranoidea dahlii*	21
P84268	Dahlein-5.2	*Ranoidea dahlii*	21
P84269	Dahlein-5.3	*Ranoidea dahlii*	20
P84271	Dahlein-5.5	*Ranoidea dahlii*	21
B3KYH5	Temporin-SHb	*Pelophylax saharicus*	50
P0DTV3	Lesueurin	*Ranoidea lesueuri*	13
P86129	Riparin-5.1	*Crinia riparia*	16
C0HJK1	U1-poneritoxin-Dq1c	*Dinoponera quadriceps*	9
C0HK83	Magainin-B1	*Xenopus borealis*	21
P84265	Dahlein-4.2	*Ranoidea dahlii*	23
P84266	Dahlein-4.3	*Ranoidea dahlii*	23
P84264	Dahlein-4.1	*Ranoidea dahlii*	23
P69033	Splendipherin	*Ranoidea splendida*	25
